# Diabetes in Sub-Saharan Africa: Kenya, Mali, Mozambique, Nigeria, South Africa and Zambia

**DOI:** 10.4103/0973-3930.45268

**Published:** 2008

**Authors:** Mario Azevedo, Sridevi Alla

**Affiliations:** Department of Epidemiology and Biostatistics, College of Public Service Chair, School of Health Sciences, College of Public Service, Jackson State University, Mississippi, USA; 1 Consultant Epidemiologist, STD/HIV Bureau, Mississippi State Department of Health, Jackson, Mississippi, USA

**Keywords:** Amputations, diabetes, hyperglycemia, insulin, neuropathy, tropical diabetes, world diabetes foundation

## Abstract

Until a few years ago, a limited number of epidemiologists or public health experts mentioned the words “diabetes.” As new lifestyles, imported dietary practices, and globalization take roots in the developing world, as Africa is, today, diabetes and its complications are considered an epidemic in Africa, compelling African governments to start paying more attention to its impact as thousands of Africans run the risk of dying young. The potential severity of diabetes is such that some epidemiologists predict that its economic impact and death toll will surpass the ravages of HIV and AIDS in the near future. On the African sub-continent, present literature and the work of the World Diabetes Foundation have highlighted three countries, namely, Mali, Mozambique, and Zambia. However, the conditions in South Africa, Kenya, and Nigeria, some of the most developed areas of the continent, provide a clue to how people are coping and how governments are responding to diabetes and its full impact. This study is, therefore, a meta-summary of the incidence and prevalence of today's emerging silent killer or diabetes in Sub-Saharan Africa. The theme is that time is running out for Africa and that, as was for HIV/AIDS, by the time the governments wake up and stop denying the catastrophic potential of the epidemic, diabetes will simply overwhelm the continent's resources, and the world will witness the death of millions of Africans. The last section is a call for action against diabetes in terms of advocacy, promotion of awareness, and public health policies that empower people to diabetes self-management.

## Introduction

The incidence of diabetes, especially type 2, is rapidly growing in the world. In 1985, an estimated 30 million people suffered with this chronic disease, which, by the end of 2006, had increased to 230 million, representing 6% of the world population. Of this number, 80% is found in the developing world.[1,2] It is estimated that, during the next 35 years, diabetic world-wise prevalence will reach 25%, with India being the hardest hit. For a long time, Africa was considered safe from many of the diseases that are called “diseases of affluence,” which plague the Western world. Similarly, there was a time when Africa was thought to be a continent, relatively free of diabetes mellitus illnesses. Today, however, diabetes is very uncommon in Africa, a situation that seemed to have remained virtually static until the 1990s and more recently.[3–6] Indeed, from 1959 to the mid-1980s, medical statistics showed that the prevalence rate of diabetes in Africa was equal to or less then 1.4%, with the exception of South Africa, where the rate was estimated to be as high as 3.6% in 2001.[7–9] But, by 1994, the continent-wise prevalence of diabetes mellitus stood at 3 million and was then predicted to double or triple by the year 2010.[10,11] Approximately, 7.1 million Africans were said to be suffering from diabetes at the end of 2000, a figure that was expected to rise to 18.6 million by 2030.[12]

As more data were made available worldwide, scientists found that the adult population of Indian descent, Africans on the continent, and their descendants in the Diaspora, and whites living in Africa, especially in South Africa and Tanzania, had the highest diabetes prevalence, respectively.[9,13,14] A few years ago, the rate of diabetes mellitus among Africans appears to have been 1%–6%; among the Caribbeans of African-descent, 10%–13% and among African Americans, 12%–15%, which is high. Interestingly, the white population in Africa has shown in the past either higher than or comparable rates to those of European whites, hovering between 6% and 10%.[15,16] The majority of the African diabetes is of type 2 (70%–90%), with only 25% showing the complications of type 1 diabetes.[16–18] Of course, despite the alarm worldwide, the situation on the continent and elsewhere among people of African descent is worsening as we write.

The racial and ethnic puzzle of diabetes has led scientists to posit that genetic predisposition might be a major factor, along with environmental factors, diet, lifestyle (inactivity), and residence.[19,20] In Africa, diabetes is more prevalent among the wealthy and the powerful, hence its designation as the “disease of opulence,” and remains more pronounced in urban areas where people tend to be less physically active, eating a diet that is rich in saturated fats and refined sugars. It is well known now that obesity is one of the most significant contributors to increased prevalence of diabetes, leading to the use of the word “diabesity,[21] both in rural and urban areas. In comparison to the rural areas, the urban setting also presents an increased prevalence of obesity.[22–26] In Africa, in general, the Word Health Organization (WHO) estimates that more than one-third of the women are obese compared to one-fourth of the men, with the poor being as vulnerable as the rich.

While some scientists are less speculative, others claim that there is strong evidence for the role played by genetic and immunological factors in diabetes pathogenesis. For Africans and their descendants, some have noted that patients of Sub-Saharan African origin who reside in diverse geographical environments (or the African Diaspora) could potentially contribute to the understanding of the genetic and environmental mediators of both diseases (type 1 and type 2).[27,28] The diabetes pandemic, which previously consisted mainly of type 2, has evolved in association with rapid cultural changes, an aging population, increasing urbanization, dietary lifestyles, and behavioral patterns without prevention and control preparedness. However, many intrinsic, individual and societal obstacles, such as poor education and illiteracy, low socioeconomic status, and lack of access to health care make uncertain the translation of diabetes research in Sub-Saharan Africa.[16,17,29]

## Diabetic Diseases

Diabetes or diabetes mellitus is defined as a “metabolic disorder caused by different factors characterized by a chronic high level of blood sugar with disturbances to carbohydrate, fat, and protein metabolism resulting from defects in insulin secretion, insulin action, or both.[30] Scientists have divided diabetes into three different types: Type 1 diabetes mellitus or insulin dependent diabetes mellitus (IDDM) or type 1 diabetes, is also known as juvenile onset diabetes. Type 2 diabetes mellitus (noninsulin dependent diabetes mellitus (NIDDM), or type 2 diabetes – adult-onset diabetes) is found in individuals who are insulin-resistant and who usually have relative insulin deficiency. Gestational diabetes mellitus (GDM), the third type, is defined as any degree of glucose intolerance with onset or first recognition during pregnancy. Recently, diabetologists have added a fourth category, tropical diabetes, suggested first in 1907, but made popular by Hugh Jones (J-type diabetes) during the mid-1950s by his study of 13 Jamaican patients.[31,32] However, tropical diabetes mellitus is less than 1% of the diabetes cases in Africa and is thought to be related to malnutrition.[10] Plasma glucose estimation remains the basic diagnostic criterion for the establishment of disease in patients. In our interviews with the endocrinologists at Kenyatta National Hospital in Nairobi, Kenya, Aga Khan Hospital in Mombasa, and the Ministry of Health, we learned that WHO guidelines are followed for the diagnosis and treatment of diabetes in the country.

## Diabetes complications and treatment: Sub-Sahara's challenges

Cardiovascular disease (CVD) is the most significant cause of death in the diabetic population.[33–37] Among persons with diabetes, part of the increased likelihood of cardiovascular disease seems to be a consequence of the increased frequency of such risk factors as hypertension, high lipids in the blood, and physical inactivity.[36,37] Diabetic retinopathy is a leading cause of adult blindness. Diabetics are six times more prone to cataracts and 1.4 times more susceptible to open-angle glaucoma when compared to the general population.[38–40] Diabetic neuropathy may present with no symptoms or pain, sensory loss, weakness, or autonomic dysfunction.[41] The condition may result in significant morbidity and may contribute to other major complications, such as lower extremity amputation, which is the major debilitating complication.[42–44] Several studies in Kenya have suggested that the prevalence of foot ulcers was found to be a significant complication at tertiary clinics like Kenyatta National Hospital.[44–46] The risk factors attributed to the ulcers were poor glycemic control, diastolic hypertension, infection, dyslipidemia, and poor self-care, which are modifiable and manageable.[46,47] Diabetic ketoacidosis (DKA) develops when absolute insulin deficiency and an absolute increase in contra-insulin hormones, increasing hepatic glucose production, decreasing peripheral glucose utilization, and stimulating release of fatty acids from fat cells and production of ketones by the liver are present.[48,49]

A study conducted at Kenyatta National Hospital found that diabetic ketoacidosis occurred in 8% of the hospitalized diabetic patients, and almost 29.8% of the patients died within 48 hours of presentation.[48] A study in Tanzania, Kenya's southern neighbor, showed that 50% of the deaths in the patients requiring insulin were due to diabetic ketoacidosis. Others studies have also shown that the complications and associated morbidity, as well as mortality, can be lowered by strict glycemic control.[47,50] In Africa, infection and acute metabolic complications have been reported as the most common causes of death in the diabetic population, in contrast to Western countries where renal and cardiovascular complications are considered to be the common causes.

Even though the prevalence of such infectious diseases as HIV/AIDS, malaria, and tuberculosis is galvanizing the health economy, diabetes seems to be the world's most threatening epidemic, which is beginning to submerge the developing world. While, on the one hand, the targeted population for diabetes includes those with 40-70 years of age, HIV/AIDS engulfs people aged between 20 and 40 years, on the other. Shortage of medicines and their high cost are key indicators of the economic capabilities of the health care system in any given country.[51–53] Most of the diabetic patients using ambulatory care in Africa do not reach the desired glycaemic level of control.[47,50] Perennial inaccessibility to insulin and its high cost are considered the major reasons behind the poor glycaemic control. For normal blood sugar, insulin is underutilized in the Third World due to culturally based misconceptions and chronic shortages.[53–55] The morbidity and mortality resulting from such inaccessibility is reflected in the younger patient population, which needs it for treatment.[55] However, contradictory results were found in such countries as Mozambique and Zambia, where the lack of tools and infrastructure, along with inadequate training of the health workers, increased the risk of misdiagnosis and failure to detect diabetes.[54]

The World Diabetes Foundation (WDF), the largest British philanthropic organization dedicated to the elimination of diabetes, has developed the so-called rapid assessment protocol for insulin access (RAPIA), a method that also determines the incidence and prevalence of diabetes in a given population. RAPIA has allowed the WDF to collect major data bases in Sub-Saharan Africa, especially in Mali, Mozambique, and Zambia.[56] However, some studies of diabetic patients in Africa have reported that the major concern in patient care is the lack of follow up.[41,47,49,52] Unfortunately, the vast majority of the cases have been diagnosed at the complications stages and not earlier.

## Diabetes education and awareness in Sub-Saharan Africa

The International Diabetes Federation (IDF) is working on standardized education in eight African countries – Tanzania, Cameroon, Mozambique, Kenya, Uganda, Senegal, Zimbabwe, and Zambia. One of the major economic burdens for these countries comes from the high prevalence of tuberculosis, malaria, and HIV/AIDS. IDF is also working on training programs in French, English, Portuguese, and Swahili for diabetes awareness and patient's self-management and care. The other major threat, which is prevalent in Sub-Saharan Africa, lies in the health belief system of patients, which leads them to rely on traditional medicine rather than “allopathic medicine.”[53,56,57] The problem with traditional healers (TH) regarding diabetes is that they rarely refer patients to public health facilities because of their little understanding of the disease, even though they may be aware of the patient's excessive thirst and frequent urination. Table 1 shows the practice of interviewed traditional healers on diabetes referrals in the three countries served by IDF.

**Table 1:** Traditional healers in Mali, Mozambique, and Zambia

Country	% of TH members in National Associations	TH Who Refer Patients to Biomedicine		
		
		Always	Sometimes	Never
Mali	83	28%	44%	28%
Mozambique	60	20%	80%	0%
Zambia	89	16%	74%	10%

Source: IDF, 2006:24.

The long window period between the precipitation of symptoms due to hyperglycemia and the beginning of undiagnosed metabolic derangement (insulin resistance, glucose tolerance, and hyperinsulinemia) necessities the intervention of education programs. Diabetes education in Kenya, to name one country where awareness is growing, was started as a joint four-year partnership of the World Diabetes Foundation (WDF), the Ministry of Health, and the Kenya Diabetes Management and Information Center.[58] When a survey consisting of 10 questions on diabetes was administered to 1,700 people in the streets of Nairobi recently, only 20% of the respondents had 70% of the questions correctly answered.[59] The results underscored the great need for diabetes education programs, which the government is attempting to implement.

Properly motivated paramedical staff is the key to providing continuing care and follow-up of diabetic patients. So far, in some developing countries, the existence of informed physicians and a high cadre of diabetes educators, nutritionists, and physiologists, have had a major impact on arresting the growing diabetes prevalence.[57] During the 19th World Diabetes Congress, the “Diabetes Declaration and Strategy for Africa” was launched by the International Diabetes Federation Africa Region. Through this declaration, IDF, WHO Africa Region, and the African Union agreed to combine their efforts and called on governments, stakeholders, and partners to work towards prevention, improve the quality of life, and reduce morbidity and premature mortality caused by the disease.[60,61] The resulting strategic planning provides mission, vision, and motivation for the attainment of goals and agreed principles.

## Discussion

Chronic diseases are now the major causes of death and disability globally. According to the World Bank, 72% of deaths due to chronic diseases occur in low-income countries. Regrettably, these countries bear the dual burden brought about by infectious and chronic diseases. Diabetes is undoubtedly for them a public health concern epidemiologically and economically. It accounts for 3.8 million deaths worldwide per year, a number similar in magnitude to the mortality attributed to HIV/AIDS.[59] Studies suggest that these deaths can be prevented, especially in economically productive individuals between the ages of 35 and 64 years of age.[1,62] Currently, however, statistics show that, every 10 minutes, someone dies from a diabetes-related disease. Unfortunately, the resources and responses to meet this epidemic have not kept pace with its demographic spread and impact. Therefore, African, as well as other countries in the world, must redouble their efforts to ensure follow-up of patients, whenever treatment has commenced and thus help reduce and/or prevent the high death toll from this chronic and debilitating disease.

It is clear that the multi-factorial risk of diabetes, which includes socioeconomic status, sedentary lifestyle, diet, and genetic patterns, must be viewed from a broader perspective. Thus, the authors, as many experts have stressed, recommend the use of a comprehensive and integrated approach involving strategic planning, action, and implementation. The recommended paradigm shift is critical towards the development of policies on non-communicable diseases and must integrate diabetes care into the management of the whole health care system. The African Millennium Development Goal #5, “Situation Analysis,” also stresses the paucity of data in Kenya's health care system and the need for accelerated accurate data collection and analysis.[63]

Even though mass screenings are not recommended, patients with risk factors should be constantly encouraged to submit to the screening process. The said fact is that the average number of visits for patient care in the diabetic population in Sub-Saharan Africa is low and usually occurs only when complications occur.[14,47] For example, in 2006, Eric Mongola, Chairman of the Kenya Diabetes Association, led the campaign “World Diabetes Day” using the slogan “Diabetes Care for Everyone.” The main objectives of the campaign have included promotion of diabetes awareness among the population, fund-raising from non-government organizations to combat non-communicable diseases, sharing of the best evidence-based interventions, involvement of network groups and individuals in the campaign, and stimulating the mission and vision of diabetes prevention.

A disproportionate increase in the numbers of diabetic patients (expected to double in the next few years) has a definite negative impact on the government's ability to treat diabetic complications, especially in the developing countries. Therefore, research on alternative sources of cure, such as plant extracts, can help African governments to improve the effort towards the prevention of diabetes. Overall, diabetes cases are expected to rise in Africa by 90% by 2010, and experts note that it will affect mostly those in the working age.[1,59] In so far as access to diabetes care in countries such as Mozambique, Mali, and Zambia, only 15% of the population have the privilege; 35% of the diabetics have difficult accessing it; and 50% have never been diagnosed.[56] Interesting are comparative figures for insulin care for people with diabetes in the three countries, as shown below.

Few statistical data are available for other African countries. In Kenya, however, official statistics note a diabetes prevalence of 3.5%, but the government itself believes the rate is much higher, about 10% of the population. The table below illustrates the state of diabetes diagnosis in the three countries served by the World Diabetes Foundation.

All over Africa, patients struggle to buy syringes to self-administer insulin. While in Mali, for example, the cost of a syringe is between US$0.20 and US$0.60, in Mozambique it is about $0.04-$0.20, and in Zambia $.01-$1.50. Urine glucose test in Mali, where the per capita GDP is $900, costs about $0.89, and blood glucose test can be performed at $2.38. Testing, insulin, and transport for a city dweller in Mali cost approximately $21.24 a month for diabetes care alone, or $225 a year, of which half, or $11 a month, would be reserved for just one vial of 100 IU insulin. While in Mozambique, the tests are free for labor inpatient members but about $0.21 for outpatients, in Zambia the cost can be as high $51.06 a month.[54,56] In Kenya, where the per capita GDP is estimated at $1,200 a year, most diabetics, even with government subsidies, are still unable to afford diabetes health care.

As noted, some governments in Africa subsidize the cost of diabetic heath care. However, as in Nigeria, where the patient still has to pay 72% of the cost on drug prescription, the most expensive aspect of the problem stands at 62%, followed by transportation and laboratory tests, which run about 7% of the total cost to the individual.[64] While in the Africa region of the World Diabetes Federation, none of the countries has a 100% access to insulin for its people, in the Democratic Republic of the Congo, where the population shows a high rate of type 1 diabetes, insulin is less than 25% of the times accessible [Table 2]. In Zambia, type 2 diabetics can only access insulin 26%-49% of the times.[11] The major reasons presented for the nonaccessibility factor are insulin being too expensive, lack of availability in all regions, transportation difficulties, high demand and lack of supply, and its “very poor quality.” A few years ago, the Gambian government was spending almost 4% of its annual expenditures only for diabetes, but that is an exception in Africa. The following table provides an idea of the various aspects of diabetes-related health care in the three countries [Tables 3, 4].

**Table 2:** Criterion for comparison of health systems for care of insulin-requiring diabetes

Countries	National prevalence	Prevalence/100,000	Availability of insulin to public sector	Average insulin cost in public sector	Availability of test materials at public facilities
Mali	3.9	3.8	17%	$10.88	43%
Mozambique	3.5	6.5	20%	$1.13	21%
Zambia	12.0	1.9	75%	$2.00	54%

Source: IDF, 2006:31.

**Table 3:** Availability of various diagnostic tools in Mali, Mozambique and Zambia at different health facilities visited

Country	# of Interventions	Presence of urine glucose strips	Presence of ketone strips	Presence of glucometer	Presence of Spectrophotometer or blood analysis equipment
Mali	30	54%	13%	43%	23%
Mozambique	37	18%	8%	21%	8%
Zambia	49	61%	49%	54%	10%

Source: IDF, 2006:21.

**Table 4:** Diabetes-related information

	Mali	Mozambique	Zambia
General lack of resources	X	X	X
Lack of control or concern for sustainability with regards to donations of medications and materials	X		
Strong political will at all levels and recognition of diabetes as a public health problem	X	X	X
WHO involvement with national centers for diabetes	X	X	X
Other NGOs working in the fields of diabetes	X	X	X
Activities for world diabetes day generate a substantial amount of interest and publicity around diabetes	X	X	X
No earmarked funds for chronic diseases/diabetes	X	X	X
Social distance between doctors and patients	X	X	X
Distribution of medicines works well at the regime/provincial level	X	X	X
No problem with cold chain	X	X	X

Source: IDF, 2006: 25.

It also often happens that even when the patient has the means to pay for insulin, which in many African countries is listed as an essential drug, the shelves are empty. While the capital city quite often is well served, the peripheral areas remain well underserved. Even in the cities, the shortages are at times common.

## Conclusions

Diabetic training, on the one hand, is one aspect that virtually all of Sub-Saharan Africa lacks. Studies have demonstrated that the lack of proper training of health professionals on diabetes accounts for the high non-compliance rates, serious complications.[14,17,58,65,66] On the other hand, the lack of national guidelines, poverty, and ignorance result in complications, such as leg amputations, which lead to gangrenes and death.[41,42] In addition, about 60% of the patients in Africa die while in treatment.[48,60] It is also known that, on the African continent foot complications are the main cause of prolonged hospital stays for people with diabetes and are associated with substantial mortality, constituting a major public health problem.[41–45] It is estimated that, in most of Africa, more than half of those suffering from diabetes die within a short interval from presentation outside the major conurbations, implying a life expectancy similar to that in Europe or North America before the insulin era.[56]

In 2002, Novo Nordisk created the World Diabetes Foundation in an attempt to make insulin more affordable to the least developed countries, lobbying for pharmaceuticals not to charge more than 20% to patients, as is their practice in the developed world. In Africa, the following countries have benefited from this organization's effort: Mauritania, Senegal, Gambia, Cape Verde, Guinea-Bissau, Guinea, Sierra Leone, Liberia, Burkina Faso, Togo, Benin, Sao Tome e Principe, Equatorial Guinea, Central African Republic, Angola, Democratic Republic of Congo, Mali, Niger, Chad, Sudan, Eritrea, Djibouti, Ethiopia, Somalia, Uganda, Rwanda, Burundi, Mozambique, Malawi, Lesotho, Zambia, Comoros, Madagascar, and Tanzania.

In tandem with the WHO and the WDF, Novo Nordisk attempts to influence governments in Africa to adopt four goals, namely, 1) create national health strategies; 2) build national health care capacity; 3) promote the best possible pricing practice; and 4) provide and seek additional funding, working through several charity organizations, such as the World Diabetes Foundation. Presently, the International Diabetes Federation, Africa region, has many members, including more than 37 countries in Sub-Saharan Africa, and the islands of Madagascar and Seychelles. The organization aims at increasing and strengthening its membership in Sub-Saharan Africa and stimulate the formation of intra-regional joint diabetes research.” As a result of these efforts, for example, in 2006, the government of Cote d'Ivoire pledged that it “would launch a national program to fight diabetes, and, in June [2007], its National Institute of Public Health pledged it would boost services for diabetes,” even though patients were still waiting for action. Despite delays, it appears that several African governments are slowly heeding the call of the world and their own national organizations to accelerate their response to the diabetes pandemic threat.

In the process, training of diabetes managers, critical to the treatment and reduction of the threat, should be a priority for in Africa. The American Revision of the National Standards for Diabetes Self-Management Education (DSME) argues that a multidisciplinary approach to the healthcare of the patient is desirable but Standard 5 allows the use of a single educator (dietician, nurse, or pharmacist) as long as that educator refers patients to other experts if needed.[67,68] Both of these recommended solutions are virtually insurmountable on the continent. Studies also emphasize that diabetes is very stressful to its victims and that most of them do need some psychological counseling to embrace what has been called “a patient-centered empowerment model that fosters collaboration and builds relationships with patients when providing clinical care”.[69] However, even though experts acknowledge that “There is no ‘best’ education program or approach; programs incorporating behavioral and psychological strategies demonstrate improved outcomes.” Additional studies show that culture- and age-appropriate programs “improve outcomes and that group education is effective.”[70–72] Unfortunately, these are luxury health care requirements that many African governments are neither economically nor psychologically prepared nor able to provide to their people as diabetes is still a very low priority of the national health care agenda.

All of these national deficiencies have tremendous policy implications at the time when HIV/AIDS and TB, malaria, yellow fever, and other tropical diseases are threatening not only people's lives in Sub-Saharan Africa but also the very economic system expected to sustain them. As a result, the situation will not improve until priorities are rethought and resources re-channeled specifically to reduce the high tide of disease.
